# The Top-100 Highly Cited Original Articles on Drug Therapy for Ventilator-Associated Pneumonia

**DOI:** 10.3389/fphar.2019.00108

**Published:** 2019-02-12

**Authors:** Chao-Yang Wang, Bing-Hui Li, Lin-Lu Ma, Ming-Juan Zhao, Tong Deng, Ying-Hui Jin, Xue-Qun Ren

**Affiliations:** ^1^Department of General Surgery, Huaihe Hospital of Henan University, Kaifeng, China; ^2^Institute of Evidence-Based Medicine and Knowledge Translation, Henan University, Kaifeng, China; ^3^Center for Evidence-Based and Translational Medicine, Zhongnan Hospital of Wuhan University, Department of Evidence-Based Medicine and Clinical Epidemiology, The Second Clinical College of Wuhan University, Wuhan, China; ^4^Center for Evidence-Based Medicine, Henan University of Chinese Medicine, Zhengzhou, China; ^5^Department of Cardiology, The First Affiliated Hospital of Henan University, Kaifeng, China

**Keywords:** bibliometrics, ventilator-associated pneumonia, drug therapy, antibiotics, VOSviewer

## Abstract

**Background:** In recent decades, research on drug therapy for ventilator-associated pneumonia (VAP) remains one of the major hot-spots in the field of critical care medicine, but relevant data are not satisfactory. Our aim was to assess the status and trends of the most cited articles on drug therapy for VAP through bibliometric approaches.

**Methods:** The Institute for Scientific Information (ISI) Web of Science core collection database was searched for the VAP-related articles. The time period for retrieval was from the beginning of the database to September 30, 2018. The top 100 most cited articles were selected to obtain their information on the authors, title, publication, number of citations, author’s affiliations, country, etc. These general information and bibliometric data were collected for analysis. VOSviewer software was used to generate a term co-occurrence graph that visualized a reference pattern for different terms in the 100 articles.

**Results:** The number of citations for the 100 selected articles ranged from 142 to 3,218. These articles were published in 31 different journals. The top three journals in terms of the number of our selected articles they published were “*Critical Care Medicine*” (17 articles), “*American Journal of Respiratory and Critical Care Medicine*” (11 articles) and “*Clinical Infectious Diseases*” (10 articles). The most frequently nominated author was Marin H. Kollef from the University of Washington, and of the top 100 articles, 16 listed his name. These top 100 articles were published after the year of 2000. The most common type of article in the top 100 was an original article (53%). The United States and France were the countries that contributed the most articles to the top 100. Gram-negative bacilli, pseudomonas aeruginosa, antibiotics, risk factors and other terms appeared more frequently, suggesting that attentions on this issue currently focused on the rational application and management of antibiotics.

**Conclusion:** This study analyzed the 100 most cited articles on drug-treated VAP, and provided insights into the historical developments and characteristics of the most cited articles in the field of VAP.

## Introduction

Ventilator-associated pneumonia (VAP) is a special form of hospital-acquired pneumonia, and is a common complication of ventilator use in intensive care units. This condition is also associated with increased mortality, extended hospital stays, and additional medical costs (Morehead and Pinto, 2000; Barbier et al., 2013; Sandrock and Shorr, 2015). According to earlier research (Vincent et al., 2009), the overall prevalence of ICU infection was 51% (7087/13796), and the usage rate of antibacterial drugs was 71%; meanwhile, 56% of ICU patients received mechanical ventilation, and the infection rate among them was 67.6%. Over the years, the incidence of VAP and the risk of VAP-related death have been reduced, with a recent estimate of 9–13%, mainly due to the implementation of prevention strategies (Melsen et al., 2013; Ding et al., 2017). Studies have shown that the total costs of hospitalization for patients with VAP are twice those of patients without VAP (Restrepo et al., 2010). Given the enormous burdens caused by this disease and its resulting mortality, many studies have focused on its accurate diagnosis, treatment and prevention around the world.

The outcome of drug therapy for VAP mainly depends on the application of antibacterial drugs. The pathogens of VAP are complex and diverse, and drug resistance appears quite frequently. Demands for antibacterial drugs are high, but serious unreasonable applications also simultaneously emerge in drug uses. According to relevant statistics, among the applications of antibacterial drugs in the ICU, 30–60% are unnecessary, inappropriate or undesirable, and about 50% of the ICU antibiotic prescriptions are for the treatment of VAP (Luyt et al., 2014). A statistical analysis by the Johns Hopkins University School of Medicine in the United States showed that patients without VAP or other infections received needless antibiotics treatments of 1 183 days during the study period (Nussenblatt et al., 2014). Proper use of antibacterials will reduce hospital stays and hospitalization costs.

Bibliometric analysis is a method assessing the status and trends of a particular research field and thus providing ideas and directions for future research. Bibliometric studies have offered insights for some special conditions, including respiratory disease (Seriwala et al., 2015), coronary heart disease (Liao et al., 2016), and hypertension (Oh and Galis, 2014). However, there has been no comprehensive review of studies on drug treatment for VAP using bibliometric methods.

The purpose of this study was to use bibliometric methods to analyze the 100 most cited articles on drug therapy for VAP, hoping to gain a deeper understanding on research status through analyzing their publication year, author, affiliations, country, journal and other key features.

## Materials and Methods

### Data Sources

The most cited VAP-related articles were retrieved from the Institute for Scientific Information (ISI) Web of Science core collection database from the beginning of the database to September 30, 2018 (updated to November 1, 2018), at Henan University, Kaifeng, Henan. VAP, drug therapy, and antibiotics were used as search terms. Articles were sorted in descending order according to the number of times cited, and 100 articles most cited were determined.

### Data Extraction

Two independent researchers evaluated each identified article to ensure that the article involved drug therapy for VAP, regardless of the article type. The following information was collected from the included articles: authors, year of publication, title, journal, article type, author’s affiliations, country, citation frequency.

### Statistical Analysis

Microsoft Excel 2013 software was used for descriptive statistical analysis of author’s affiliations, country, journal, and the number of citations; VOSviewer 1.6.8 software (van Eck and Waltman, 2010) was used for visual analysis, mapping network diagram of keyword co-occurrences and co-authored researchers. In the terms’ map, each circle represented a term. The size of the circle indicated how often it appeared. The color of a circle indicated the average number of citations received. If they appeared together in any of the 100 articles, one line connected the two circles, and the thicker the line was, the more times they appeared together. If two terms appeared more frequently together, two circles would be closer. The term map visualization appears in at least five times of the 100 articles.

## Results

The 100 most-cited manuscripts were mainly original articles (53%) and reviews (38%), and few of them were proceedings papers (9%). The total number of citations for each article ranged from 142 to 3,218, and the mean number of citations was 351.82. Nearly one-third of articles (*n* = 30) showed more than 300 citations, and only five articles were cited more than 1,000 times. These top 100 most-cited articles were published between the years of 2000 and 2016. The most recent one was published in September 2016 with 367 citations (20th in the Top 100), and functions as a clinical practice guide for the management of hospital-acquired and VAP in adults from the American Society of Infectious Diseases and the American Thoracic Society, which was published by Kalil et al. (2016) in *Clinical Infectious Diseases*. Supplementary Table S1 shows the data for the top 100 cited articles evaluated in the present study.

**FIGURE 1:**
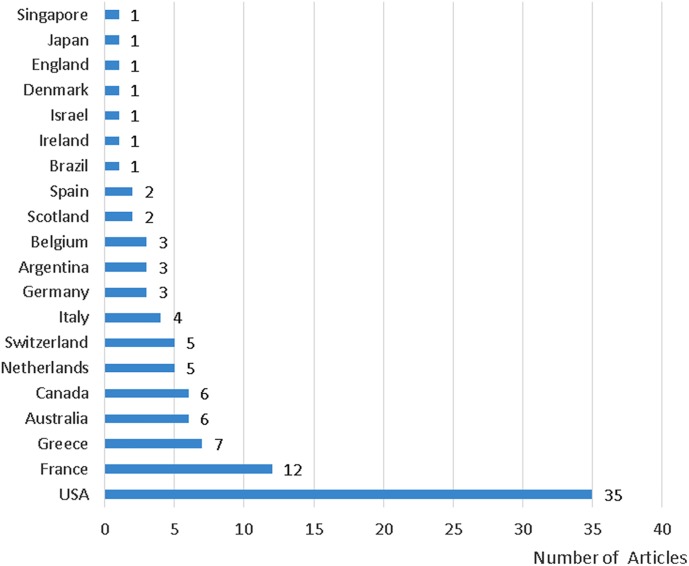
Countries from which the 100 most-cited articles originated and the number of our collected articles from each of those countries.

The top 100 articles are from 20 countries. Only the United States (*n* = 35) and France (*n* = 12) contributed more than 10 articles, followed by Greece (*n* = 7), Australia (*n* = 6), Canada (*n* = 6), and the Netherlands (*n* = 5) and Switzerland (*n* = 5). The countries from which the Top 100 article originated and the number of our collected articles, from each of those countries are shown in Figure 1.

The University of Washington produced 32% of the top 100 articles, followed by The University of Queensland (6%), Canada’s McMaster University (5%), Australia’s Royal Brisbane and Women’s Hospital (5%) and Tufts University (5%) (Table 1).

**Table 1:** Institutions contributing to the 100 most-cited articles.

Institution name	Number of articles
Washington University	32
The University of Queensland	6
McMaster University	5
Royal Brisbane and Women’s Hospital	5
Tufts University	5
Queens University	4
The Alfa Institute of Biomedical Sciences	3
Harvard University	3
Henry Dunant Hospital	3
Bichat-Claude Bernard Hospital	3


The top 10 authors most listed in the top 100 articles are summarized in Table 2. The list was led by Marin H. Kollef from the University of Washington’s St. Louis School of Medicine, who wrote 16 of the top 100 articles; meanwhile, three other authors in the list also came from the same university, two authors following Marin H. Kollef were from the University of Queensland. All of the hospitals in the list are teaching hospitals.

**Table 2:** Top 10 authors most frequently appearing in the articles.

Author	Number of articles	Affiliation	Country
Kollef MH	16	Washington University	United States
Fraser V	7	Washington University	United States
Chastre J	6	Groupe Hospitalier Pitié-Salpêtrière	France
Roberts JA	6	University of Queensland	Australia
Ward S	5	Washington University	United States
Lipman J	5	University of Queensland	Australia
Sherman G	4	Washington University	United States
Muscedere J	4	Queens University	Canada
Falagas ME	4	Alfa Institute of Biomedical Sciences	Greece
Fagon J	3	Hôpital européen Georges-Pompidou	France


The top 100 articles were published in 31 journals with influence factors ranging from 0 to 79.258 (mean ±± SD: 13.324 ± 15.562). Half of the 100 articles were published in journals of critical care (*n* = 41) or infectious diseases (*n* = 24). The journals (Table 3) which published the top 100 articles are led by *Critical Care Medicine* (*n* = 17), *American Journal of Respiratory and Critical Care Medicine* (*n* = 11), *Clinical Infectious Diseases* (*n* = 10) and *Chest* (*n* = 8). These are top journals in the field of critical care and infectious disease.

**Table 3:** Journals of the 100 most-cited articles.

Journal	No. of articles	Citation count	Country	Impact factor	Quartile in category^$^
*Critical Care Medicine*	17	3,671	United States	6.630	Q1
*American Journal of Respiratory and Critical Care Medicine*	11	7,511	United States	15.239	Q1
*Clinical Infectious Diseases*	10	6,048	United States	9.117	Q1
*Chest*	8	2,940	United States	7.652	Q1
*Antimicrobial Agents and Chemotherapy*	4	1,660	United States	4.255	Q1
*Annals of Internal Medicine*	4	1,496	United States	19.384	Q1
*Lancet*	3	1,264	ENGLAND	53.254	Q1
*New England Journal of Medicine*	3	1,251	United States	79.258	Q1
*Lancet Infectious Diseases*	3	748	United States	25.148	Q1
*Clinical Microbiology Reviews*	3	719	United States	20.642	Q1
*Journal of Antimicrobial Chemotherapy*	3	641	ENGLAND	5.217	Q1
*Clinical Pharmacokinetics*	3	543	NEW ZEALAND	4.464	Q1
*Critical Care*	3	533	ENGLAND	6.425	Q1
*International Journal of Antimicrobial Agents*	3	529	NETHERLANDS	4.253	Q1
*American Journal of Infection Control*	2	509	United States	1.929	Q3
*Clinical Microbiology and Infection*	2	507	ENGLAND	5.394	Q1
*Journal of Hospital Infection*	2	470	ENGLAND	3.354	Q2
*Expert Review of Anti-Infective Therapy*	2	361	ENGLAND	3.141	Q2
*European Respiratory Journal*	2	329	ENGLAND	12.242	Q1
*Jama-Journal of the American Medical Association*	1	711	United States	47.661	Q1
*Nature Reviews Microbiology*	1	536	ENGLAND	31.851	Q1
*American Journal of Medicine*	1	404	United States	5.117	Q1
*Fems Yeast Research*	1	317	ENGLAND	2.609	Q2
*Molecular Microbiology*	1	242	United States	3.816	Q2
*Intensive Care Medicine*	1	200	United States	15.008	Q1
*British Medical Journal*	1	199	ENGLAND	23.259	Q1
*Proceedings of the National Academy of Sciences of the United States of America*	1	190	United States	9.504	Q1
*Journal of Critical Care*	1	178	United States	2.872	Q2
*Plos One*	1	157	United States	2.766	Q1
*Current Opinion in Infectious Diseases*	1	162	United States	3.782	Q2
*Archives of Internal Medicine*	1	156	United States	8.762^#^	Q1^#^


*^$^Data from the 2017 edition of Journal Citation Reports. ^#^Data from the 2014 edition of Journal Citation Reports*.

**FIGURE 2:**
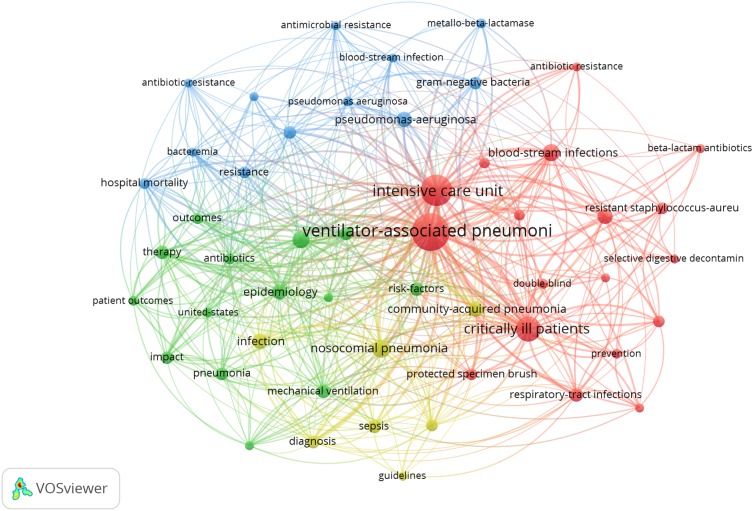
Network visualization using words from the titles and abstracts of the 100 most-cited articles.

Fifty-one terms or phrases appeared five times or more in the titles or abstracts of the top 100 articles (Figure 2). For example, “VAP” appeared 81 times, “intensive care unit” 56 times, “critically ill patients” 37 times, “nosocomial pneumonia” 19 times, while “mortality” and “blood-stream infections” 17 times. Accordingly, the terms or phrases associated with VAP are divided into four clusters, represented by four colors (red, green, blue and orange). As shown in Figure 2, many terms may reflect VAP-related content, such as: epidemiology, Gram-negative bacilli, infection, community-acquired pneumonia, pseudomonas-aeruginosa, etc. From these terms, we could find that the current attentions of drug therapy for VAP mainly focus on clinical application of antibacterial drugs, the types of pathogenic bacteria, epidemiological investigations and the standardization of clinical treatment. The 20 terms or phrases that appeared most frequently are listed in Table 4. After scoring the average publication year of the documents in which a keyword or a term occurs, we found some terms, like pseudomonas-aeruginosa bacteremia, Gram-negative bacteria, beta-lactam antibiotics, metallo-beta-lactamase, blood-stream infections becomes the hotspots in the field of VAP.

**Table 4:** Top 20 terms with most frequent occurrence in the titles or abstract.

Keywords	Occurrences	Total link strength
Ventilator-associated pneumonia	81	394
Intensive care unit	56	310
Critically ill patients	37	205
Nosocomial pneumonia	19	102
Mortality	17	108
Blood-stream infections	17	74
Epidemiology	14	92
Gram-negative bacilli	14	71
Infection	13	85
Community-acquired pneumonia	13	62
Pseudomonas-aeruginosa	13	53
Therapy	11	80
Sepsis	11	62
Mechanical ventilation	10	67
Inadequate antimicrobial treatment	10	65
Respiratory-tract infections	10	52
Risk-factors	10	52
Diagnosis	9	63
Nosocomial infections	9	62
Impact	9	58


## Discussion

In this study, we identified and analyzed the 100 most-cited articles in the field of drug therapy for VAP. The purpose of this bibliometric analysis was to assess the status and trends of the most-cited articles in the field of drug therapy for VAP.

Most of the 100 articles were from European countries and the United States, and only three were from Asian countries. Possible reason for this phenomenon may be that the definition of VAP is slightly different between countries or organizations (Kalil et al., 2016; Torres et al., 2017). Currently, these has been no gold standard for VAP diagnosis. The causes of this condition in different regions and hospitals are different, and the bacterial spectrum also varies between patients and ICUs. Up to now, VAP is usually diagnosed through combining the result from positive bacterial culture with patients’ clinical symptoms, and antibiotics for treatment would be determined based on the results of drug susceptibility tests (Moreira et al., 2013; Patro et al., 2018).

The number of citations for the Top 100 articles ranged from 142 to 3,218. Compared with other specialties (Oh and Galis, 2014; Seriwala et al., 2015; Liao et al., 2016), the number of citations was not large, probably because our research topic only involves a part of the respiratory system and critical care. However, considering physical, psychological and economic burdens from VAP on patients, study on drug therapy for VAP is also very important.

The Top 100 articles were published in 31 journals. Among the journals, 2 core ones published 28 papers, in addition, 26 articles were published in 4 journals, 24 articles were published in 8 journals and 22 articles were published in 17 journals. This journals are sorted by number of articles into four groups, each with about quarter of all articles (28:26:24:22), then the number of journals in each group will be proportional to1:2:2^2^:2^3^ (2:4:8:17). This distribution of the publications was consistent with Bradford’s law (Bradford, 1985). Ninety percent of the top 100 articles were published in journals of the Q1 from Journal Citation Reports 2017 edition. These journals are generally the top ones of the profession or the medicine field. These journals with high impact factor attract higher quality papers, and their publications of excellent articles can further elevate their academic influence in turn (Callaham et al., 2002).

According to the results on keyword co-occurrence, the top 100 most-cited articles covered a wide range of topics, including all aspects of drug-treated VAP, such as VAP risk factors (Falagas and Kopterides, 2006), pathogen production, VAP prevention (Muscedere et al., 2008), diagnosis (Fartoukh et al., 2003), treatment using antibiotics (Barlam et al., 2016), rational use of antibiotics (Bouadma et al., 2010), mortality (Bercault and Boulain, 2001), the investigation on VAP epidemics (Koeman et al., 2006), and the burden of the disease (Restrepo et al., 2010). Patients in ICU always have low immunity, suffering endogenous infections; meanwhile, VAP pathogens are highly resistant to antibiotics (Zavascki et al., 2010). Studies (Lisboa et al., 2008; Ramirez et al., 2008) have found that C-reactive protein (CRP) and procalcitonin (PCT) can be used as screening methods for VAP, and that when combining PCT with Clinical Pulmonary Infection Scores (CPIS), the diagnostic specificity for VAP can reach 100%. At the same time, the decline of CRP is closely related to the application of appropriate antibiotic treatment. Therefore, VAP prevention, early diagnosis and appropriate treatment measures are key to reduce the mortality and medical burden. High-quality training of ICU medical staffs to strengthen preventive measures for VAP-related pathophysiology can effectively reduce the occurrence of complications. In recent years, a practitioner of critical care medicine can focus on the treatment of Gram-negative bacteria, such as Pseudomonas aeruginosa, by combined use of antibiotics, and explore the mechanism of antibiotic resistance acquisition.

We acknowledge that this study has some limitations. We only searched the ISI Web of Science core collection database, with other databases such as PubMed and Scopus unsearched, and the final results were also affected by the search strategy and inclusion/exclusion criteria. Some of influential articles may be missed in our bibliometric analysis. Since the citation frequency of an article is influenced by many factors, such as the influence of journals, authors, institutions, etc., the academic influence of articles cannot be reflected only by their cited times. In addition, the citation frequency of earlier published articles should be more than that of recently published ones (Kollef et al., 2017), though academic influence of former ones may be not necessarily heavier than that of latter ones. Hence, academic impact should be simultaneously adjudged for multiple parameters, such as H-index and citation density. There were also publication bias and/or language biases in our research. Despite these limitations, we did provide insights into the development and characteristics of drug therapy for VAP.

## Conclusion

In this study, we analyzed the top 100 most-cited articles on drug therapy for VAP via bibliometrics. These top 100 articles were published after the year 2000 in top journals in the field of critical care medicine and respiratory systems. Clinical trials and antibacterial usage are the main topics of these articles, which contribute to the development and optimization of antibiotics’ application. The United States and France produced the most highly cited papers in VAP. Prof Marin H. Kollef was the author of the most papers in the top 100 in VAP. This report provides insights into historical developments and characteristics of the most-cited articles on drug therapy for VAP.

## Author Contributions

Y-HJ and X-QR designed this study. C-YW and B-HL performed the search and collected data, L-LM re-checked data. C-YW and M-JZ performed analysis, TD re-checked. C-YW and B-HL wrote the manuscript, Y-HJ and X-QR reviewed the manuscript.

## Conflict of Interest Statement

The authors declare that the research was conducted in the absence of any commercial or financial relationships that could be construed as a potential conflict of interest.

## Supplementary Material

The Supplementary Material for this article can be found online at: https://www.frontiersin.org/articles/10.3389/fphar.2019.00108/full#supplementary-material
