# Supplementary material for: The Top-100 Highly Cited Original Articles on Drug Therapy for Ventilator-Associated Pneumonia
Source: Front Pharmacol. 2019 Feb 12;10:108. doi: 10.3389/fphar.2019.00108 (PMC6379351; doi:10.3389/fphar.2019.00108)
Supplement: Supplementary file 1 [file Table_1.docx]

***Supplementary Material***

**The Top-100 Highly Cited Original Articles on drug therapy for ventilator-associated pneumonia**

**Chao-Yang Wang^1,2,3^, Bing-Hui Li^1,2,3^, Lin-Lu Ma^3,4^, Ming-Juan Zhao^2,3,5^, Tong Deng^1,2,3^, Ying-Hui Jin^2,3*^, Xue-Qun Ren^1,2*^**

**^*^** **Correspondence:** Ying-Hui Jin: jinyinghuiebm@163.com; Xue-Qun Ren: renxuequn001@163.com.

**Supplementary TABLE S1** List of The top-100 highly cited original articles on drug therapy for ventilator-associated pneumonia ranked according to their total citation counts^*^

| **Rank** | **Title** | **Journal** | **Impact factor** | **Total citation count** |
| --- | --- | --- | --- | --- |
| 1 | Guidelines for the management, of adults with hospital-acquired, ventilator-associated, and healthcare-associated pneumonia | American Journal Of Respiratory And Critical Care Medicine | 15.239 | 3398 |
| 2 | Bad Bugs, No Drugs: No ESKAPE! An Update from the Infectious Diseases Society of America | Clinical Infectious Diseases | 9.117 | 2320 |
| 3 | Ventilator-associated pneumonia | American Journal Of Respiratory And Critical Care Medicine | 15.239 | 1645 |
| 4 | Infectious Diseases Society of America and the Society for Healthcare Epidemiology of America guidelines for developing an institutional program to enhance antimicrobial stewardship | Clinical Infectious Diseases | 9.117 | 1630 |
| 5 | The influence of inadequate antimicrobial treatment of bloodstream infections on patient outcomes in the ICU setting | Chest | 7.652 | 1193 |
| 6 | Delaying the empiric treatment of Candida bloodstream infection until positive blood culture results are obtained: a potential risk factor for hospital mortality | Antimicrobial Agents And Chemotherapy | 4.255 | 795 |
| 7 | Comparison of 8 vs 15 days of antibiotic therapy for ventilator-associated pneumonia in adults - A randomized trial | JAMA-Journal Of The American Medical Association | 47.661 | 711 |
| 8 | Short-course empiric antibiotic therapy for patients with pulmonary infiltrates in the intensive care unit - A proposed solution for indiscriminate antibiotic prescription | American Journal Of Respiratory And Critical Care Medicine | 15.239 | 628 |
| 9 | Clinical importance of delays in the initiation of appropriate antibiotic treatment for ventilator-associated pneumonia | Chest | 7.652 | 619 |
| 10 | Use of procalcitonin to reduce patients' exposure to antibiotics in intensive care units (PRORATA trial): a multicentre randomised controlled trial | Lancet | 53.254 | 566 |
| 11 | CURRENT CONCEPTS Hospital-Acquired Infections Due to Gram-Negative Bacteria | New England Journal Of Medicine | 79.258 | 565 |
| 12 | Antimicrobial activity of metals: mechanisms, molecular targets and applications | Nature Reviews Microbiology | 31.851 | 536 |
| 13 | Invasive and noninvasive strategies for management of suspected ventilator-associated pneumonia - A randomized trial | Annals Of Internal Medicine | 19.384 | 512 |
| 14 | Decontamination of the Digestive Tract and Oropharynx in ICU Patients | New England Journal Of Medicine | 79.258 | 428 |
| 15 | Inappropriate initial antimicrobial therapy and its effect on survival in a clinical trial of immunomodulating therapy for severe sepsis | American Journal Of Medicine | 5.117 | 404 |
| 16 | Inadequate antimicrobial treatment: An important determinant of outcome for hospitalized patients | Clinical Infectious Diseases | 9.117 | 384 |
| 17 | Pharmacokinetic issues for antibiotics in the critically ill patient | Critical Care Medicine | 6.630 | 382 |
| 18 | Pathogen-host interactions in Pseudomonas aeruginosa pneumonia | American Journal Of Respiratory And Critical Care Medicine | 15.239 | 378 |
| 19 | Implementing an Antibiotic Stewardship Program: Guidelines by the Infectious Diseases Society of America and the Society for Healthcare Epidemiology of America | Clinical Infectious Diseases | 9.117 | 375 |
| 20 | Management of Adults With Hospital-acquired and Ventilator-associated Pneumonia: 2016 Clinical Practice Guidelines by the Infectious Diseases Society of America and the American Thoracic Society | Clinical Infectious Diseases | 9.117 | 367 |
| 21 | The commonality of risk factors for nosocomial colonization and infection with antimicrobial-resistant Staphylococcus aureus, enterococcus, gram-negative bacilli, Clostridium difficile, and Candida | Annals Of Internal Medicine | 19.384 | 364 |
| 22 | Effects of selective decontamination of digestive tract on mortality and acquisition of resistant bacteria in intensive care: a randomised controlled trial | Lancet | 53.254 | 352 |
| 23 | Nosocomial infections in adult intensive-care units | Lancet | 53.254 | 346 |
| 24 | Experience with a clinical guideline for the treatment of ventilator-associated pneumonia | Critical Care Medicine | 6.630 | 344 |
| 25 | Resolution of ventilator-associated pneumonia: Prospective evaluation of the clinical pulmonary infection score as an early clinical predictor of outcome | Critical Care Medicine | 6.630 | 326 |
| 26 | Pseudomonas aeruginosa bloodstream infection: Importance of appropriate initial antimicrobial treatment | Antimicrobial Agents And Chemotherapy | 4.255 | 321 |
| 27 | Evidence-based clinical practice guideline for the prevention of ventilator-associated pneumonia | Annals Of Internal Medicine | 19.384 | 317 |
| 28 | Candida biofilms on implanted biomaterials: a clinically significant problem | Fems Yeast Research | 2.609 | 317 |
| 29 | Pseudomonas aeruginosa: resistance and therapeutic options at the turn of the new millennium | Clinical Microbiology And Infection | 5.394 | 312 |
| 30 | Antimicrobial stewardship programs in health care systems | Clinical Microbiology Reviews | 20.642 | 309 |
| 31 | Population Pharmacokinetic Analysis of Colistin Methanesulfonate and Colistin after Intravenous Administration in Critically Ill Patients with Infections Caused by Gram-Negative Bacteria | Antimicrobial Agents And Chemotherapy | 4.255 | 305 |
| 32 | Current control and treatment of multidrug-resistant Acinetobacter baumannii infections | Lancet Infectious Diseases | 25.148 | 305 |
| 33 | Antibiotic resistance in the intensive care unit | Annals Of Internal Medicine | 19.384 | 303 |
| 34 | Guidelines for the control and prevention of meticillin-resistant Staphylococcus aureus (MRSA) in healthcare facilities | Journal Of Hospital Infection | 3.354 | 286 |
| 35 | Before-after study of a standardized hospital order set for the management of septic shock | Critical Care Medicine | 6.630 | 272 |
| 36 | Type III protein secretion is associated with poor clinical outcomes in patients with ventilator-associated pneumonia caused by Pseudomonas aeruginosa | Critical Care Medicine | 6.630 | 262 |
| 37 | Microbial Etiologies of Hospital-Acquired Bacterial Pneumonia and Ventilator-Associated Bacterial Pneumonia | Clinical Infectious Diseases | 9.117 | 260 |
| 38 | A randomized trial of diagnostic techniques for ventilator-associated pneumonia | New England Journal Of Medicine | 79.258 | 258 |
| 39 | International Nosocomial Infection Control Consortium (INICC) report, data summary for 2003-2008, issued June 2009 | American Journal Of Infection Control | 1.929 | 257 |
| 40 | International Nosocomial Infection Control Consortium (INICC) report, data summary of 36 countries, for 2004-2009 | American Journal Of Infection Control | 1.929 | 252 |
| 41 | Rhamnolipids mediate detachment of Pseudomonas aeruginosa from biofilms | Molecular Microbiology | 3.816 | 242 |
| 42 | Infection control in the ICU | Chest | 7.652 | 242 |
| 43 | Oral decontamination with chlorhexidine reduces the incidence of ventilator-associated pneumonia | American Journal Of Respiratory And Critical Care Medicine | 15.239 | 240 |
| 44 | Systematic Review and Meta-Analysis of the Efficacy of Appropriate Empiric Antibiotic Therapy for Sepsis | Antimicrobial Agents And Chemotherapy | 4.255 | 239 |
| 45 | Individualised antibiotic dosing for patients who are critically ill: challenges and potential solutions | Lancet Infectious Diseases | 25.148 | 230 |
| 46 | Does antibiotic exposure increase the risk of methicillin-resistant Staphylococcus aureus (MRSA) isolation? A systematic review and meta-analysis | Journal Of Antimicrobial Chemotherapy | 5.217 | 228 |
| 47 | Rotation and restricted use of antibiotics in a medical intensive cave unit - Impact on the incidence of ventilator-associated pneumonia caused by antibiotic-resistant gram-negative bacteria | American Journal Of Respiratory And Critical Care Medicine | 15.239 | 227 |
| 48 | Prevention of ventilator-associated pneumonia by oral decontamination - A prospective, randomized, double-blind, placebo-controlled study | American Journal Of Respiratory And Critical Care Medicine | 15.239 | 224 |
| 49 | Guidelines for the prophylaxis and treatment of methicillin-resistant Staphylococcus aureus (MRSA) infections in the UK | Journal Of Antimicrobial Chemotherapy | 5.217 | 223 |
| 50 | Prevention of hospital-associated pneumonia and ventilator-associated pneumonia | Critical Care Medicine | 6.630 | 219 |
| 51 | Resolution of infectious parameters after antimicrobial therapy in patients with ventilator-associated pneumonia | American Journal Of Respiratory And Critical Care Medicine | 15.239 | 216 |
| 52 | Stenotrophomonas maltophilia: an emerging opportunist human pathogen | Lancet Infectious Diseases | 25.148 | 213 |
| 53 | Procalcitonin-guided interventions against infections to increase early appropriate antibiotics and improve survival in the intensive care unit: A randomized trial | Critical Care Medicine | 6.630 | 211 |
| 54 | The epidemiological profile of infections with multidrug-resistant Pseudomonas aeruginosa and Acinetobacter species | Clinical Infectious Diseases | 9.117 | 211 |
| 55 | Combination Therapy for Treatment of Infections with Gram-Negative Bacteria | Clinical Microbiology Reviews | 20.642 | 211 |
| 56 | Mortality rate attributable to ventilator-associated nosocomial pneumonia in an adult intensive care unit: A prospective case-control study | Critical Care Medicine | 6.630 | 204 |
| 57 | Antimicrobial therapy in critically ill patients - A review of pathophysiological conditions responsible for altered disposition and pharmacokinetic variability | Clinical Pharmacokinetics | 4.464 | 204 |
| 58 | Procalcitonin kinetics as a prognostic marker of ventilator-associated pneumonia | American Journal Of Respiratory And Critical Care Medicine | 15.239 | 202 |
| 59 | Impact of appropriateness of initial antibiotic therapy on the outcome of ventilator-associated pneumonia | Intensive Care Medicine | 15.008 | 200 |
| 60 | Oral decontamination for prevention of pneumonia in mechanically ventilated adults: systematic review and meta-analysis | British Medical Journal | 23.259 | 199 |
| 61 | New Approaches to Sepsis: Molecular Diagnostics and Biomarkers | Clinical Microbiology Reviews | 20.642 | 199 |
| 62 | A randomized controlled trial of an antibiotic discontinuation policy for clinically suspected ventilator-associated pneumonia | Chest | 7.652 | 196 |
| 63 | Treatment and control of severe infections caused by multiresistant Pseudomonas aeruginosa | Clinical Microbiology And Infection | 5.394 | 195 |
| 64 | Multidrug-resistant Pseudomonas aeruginosa and Acinetobacter baumannii: resistance mechanisms and implications for therapy | Expert Review Of Anti-Infective Therapy | 3.141 | 192 |
| 65 | Emergence of resistance to carbapenems in Acinetobacter baumannii in Europe: clinical impact and therapeutic options | International Journal Of Antimicrobial Agents | 4.253 | 191 |
| 66 | Ecological theory suggests that antimicrobial cycling will not reduce antimicrobial resistance in hospitals | Proceedings Of The National Academy Of Sciences Of The United States Of America | 9.504 | 190 |
| 67 | Guidelines for the management of hospital-acquired pneumonia in the UK: Report of the Working Party on hospital-acquired pneumonia of the British Society for Antimicrobial Chemotherapy | Journal Of Antimicrobial Chemotherapy | 5.217 | 190 |
| 68 | Clinical characteristics and treatment patterns among patients with ventilator-associated pneumonia | Chest | 7.652 | 189 |
| 69 | Procalcitonin to guide duration of antibiotic therapy in intensive care patients: a randomized prospective controlled trial | Critical Care | 6.425 | 187 |
| 70 | Efficacy and safety of intravenous infusion of doripenem versus imipenem in ventilator-associated pneumonia: A multicenter, randomized study | Critical Care Medicine | 6.630 | 187 |
| 71 | Colistin therapy for microbiologically documented multidrug-resistant Gram-negative bacterial infections: a retrospective cohort study of 258 patients | International Journal Of Antimicrobial Agents | 4.253 | 184 |
| 72 | Risk factors for the isolation of multi-drug-resistant Acinetobacter baumannii and Pseudomonas aeruginosa: a systematic review of the literature | Journal Of Hospital Infection | 3.354 | 184 |
| 73 | Pneumonia caused by methicillin-resistant Staphylococcus aureus | Clinical Infectious Diseases | 9.117 | 182 |
| 74 | Diagnosing pneumonia during mechanical ventilation - The clinical pulmonary infection score revisited | American Journal Of Respiratory And Critical Care Medicine | 15.239 | 182 |
| 75 | Comprehensive evidence-based clinical practice guidelines for ventilator-associated pneumonia: Prevention | Journal Of Critical Care | 2.872 | 178 |
| 76 | Antibacterial dosing in intensive care - Pharmacokinetics, degree of disease and pharmacodynamics of sepsis | Clinical Pharmacokinetics | 4.464 | 177 |
| 77 | Intravenous colistin in the treatment of sepsis from multiresistant Gram-negative bacilli in critically ill patients | Critical Care | 6.425 | 176 |
| 78 | Influence of combined intravenous and topical antibiotic prophylaxis on the incidence of infections, organ dysfunctions, and mortality in critically ill surgical patients - A prospective, stratified, randomized, double-blind, placebo-controlled clinical trial | American Journal Of Respiratory And Critical Care Medicine | 15.239 | 171 |
| 79 | Health-Care-Associated Pneumonia Among Hospitalized Patients in a Japanese Community Hospital | Chest | 7.652 | 171 |
| 80 | Insufficient beta-lactam concentrations in the early phase of severe sepsis and septic shock | Critical Care | 6.425 | 170 |
| 81 | Procalcitonin for reduced antibiotic exposure in ventilator-associated pneumonia: a randomised study | European Respiratory Journal | 12.242 | 170 |
| 82 | Colistin: an update on the antibiotic of the 21st century | Expert Review Of Anti-Infective Therapy | 3.141 | 169 |
| 83 | A comparative analysis of patients with early-onset vs late-onset nosocomial pneumonia in the ICU setting | Chest | 7.652 | 168 |
| 84 | Healthcare-associated bloodstream infection: A distinct entity? Insights from a large US database | Critical Care Medicine | 6.630 | 167 |
| 85 | Predictors of mortality for methicillin-resistant Staphylococcus aureus health-care-associated pneumonia - Specific evaluation of vancomycin pharmacokinetic indices | Chest | 7.652 | 162 |
| 86 | Aerosolized antibiotics and ventilator-associated tracheobronchitis in the intensive care unit | Critical Care Medicine | 6.630 | 162 |
| 87 | Augmented Renal Clearance Implications for Antibacterial Dosing in the Critically Ill | Clinical Pharmacokinetics | 4.464 | 162 |
| 88 | Colistin in the 21st century | Current Opinion In Infectious Diseases | 3.782 | 162 |
| 89 | Impact of a rotating empiric antibiotic schedule on infectious mortality in an intensive care unit | Critical Care Medicine | 6.630 | 161 |
| 90 | Colistin and Rifampicin Compared With Colistin Alone for the Treatment of Serious Infections Due to Extensively Drug-Resistant Acinetobacter baumannii: A Multicenter, Randomized Clinical Trial | Clinical Infectious Diseases | 9.117 | 160 |
| 91 | Clinical Outcomes With Extended or Continuous Versus Short-term Intravenous Infusion of Carbapenems and Piperacillin/Tazobactam: A Systematic Review and Meta-analysis | Clinical Infectious Diseases | 9.117 | 159 |
| 92 | Procalcitonin-guided algorithms of antibiotic therapy in the intensive care unit: A systematic review and meta-analysis of randomized controlled trials | Critical Care Medicine | 6.630 | 159 |
| 93 | Biomarkers in respiratory tract infections: diagnostic guides to antibiotic prescription, prognostic markers and mediators | European Respiratory Journal | 12.242 | 159 |
| 94 | A survival benefit of combination antibiotic therapy for serious infections associated with sepsis and septic shock is contingent only on the risk of death: A meta-analytic/meta-regression study | Critical Care Medicine | 6.630 | 158 |
| 95 | The Adult Cystic Fibrosis Airway Microbiota Is Stable over Time and Infection Type, and Highly Resilient to Antibiotic Treatment of Exacerbations | Plos One | 2.766 | 157 |
| 96 | Prediction of Infection Due to Antibiotic-Resistant Bacteria by Select Risk Factors for Health Care-Associated Pneumonia | Archives Of Internal Medicine | 8.762^#^ | 156 |
| 97 | Optimal management therapy for Pseudomonas aeruginosa ventilator-associated pneumonia: An observational, multicenter study comparing monotherapy with combination antibiotic therapy | Critical Care Medicine | 6.630 | 156 |
| 98 | De-escalation therapy in ventilator-associated pneumonia | Critical Care Medicine | 6.630 | 155 |
| 99 | New insights into meticillin-resistant Staphylococcus aureus (MRSA) pathogenesis, treatment and resistance | International Journal Of Antimicrobial Agents | 4.253 | 154 |
| 100 | A systematic review on clinical benefits of continuous administration of beta-lactam antibiotics | Critical Care Medicine | 6.630 | 146 |

^*^Total citation count update to November 1, 2018; The impact factor data from the 2017 edition of Journal Citation Reports; ^#^ Data from the 2014 edition of Journal Citation Reports.
